# Comparison of Health Service Utilization for Febrile Children Before and After Introduction of Malaria Rapid Diagnostic Tests and Artemisinin-Based Combination Therapy in Rural Papua New Guinea

**DOI:** 10.3389/fpubh.2018.00075

**Published:** 2018-03-13

**Authors:** Takahiro Tsukahara, Takuma Sugahara, Takuro Furusawa, Francis Wanak Hombhanje

**Affiliations:** ^1^Department of International Affairs and Tropical Medicine, Tokyo Women’s Medical University, Tokyo, Japan; ^2^Graduate School of Economics, Hosei University, Tokyo, Japan; ^3^Department of Ecology and Environment, Graduate School of Asian and African Area Studies, Kyoto University, Kyoto, Japan; ^4^Centre for Health Research and Diagnostics, Divine Word University, Madang, Papua New Guinea

**Keywords:** antimalarials, delivery of health care, health service needs and demand, treatment-seeking behavior, sex factors

## Abstract

**Background:**

In Papua New Guinea (PNG), a malaria treatment policy using rapid diagnostic tests (RDTs) plus artemisinin-based combination therapy (ACT) was widely introduced to rural communities in 2012. The objectives of the study were to evaluate the effect of this RDT/ACT introduction to a rural PNG population on health service utilization and to compare factors associated with health service utilization before and after the RDT/ACT introduction.

**Methods:**

Household surveys with structured questionnaires were conducted before and after the introduction of RDT/ACT in a catchment area of a health center in East Sepik Province, PNG. We interviewed caregivers with children less than 15 years of age and collected data on fever episodes in the preceding 2 weeks. Using propensity score matching, febrile children before the introduction of RDT/ACT were matched to febrile children after the introduction. Then, the adjusted difference in the proportion of health service utilization [i.e., the average treatment effect (ATE) of the introduction of RDT/ACT on health service utilization] was estimated. We also employed a multilevel Poisson regression model to investigate factors influencing the use of health services.

**Results:**

Of 4,690 children, 911 (19%) were reported to have a fever episode. The unadjusted proportion of health service utilization was 51.7 and 57.2% before and after the RDT/ACT introduction, respectively. After matching, no significant difference in the health service utilization was observed before and after the introduction of RDT/ACT (ATE: 0.063, 95% confidence interval −0.024 to 0.150). Multilevel regression analysis showed that the consistent factors associated with a higher utilization of health services were severe illness and being female.

**Conclusion:**

The utilization of health services was not significantly different before and after the introduction of RDT/ACT. Villagers may have neither sufficient informations on the new protocol nor high acceptance of RDT/ACT. The observed gender bias in health service utilization could be due to female caregivers’ preferences toward girls.

## Introduction

Despite the recent progress of investments in global malaria control, an estimated 212 million malaria cases and 429,000 malaria deaths still occurred in 2015 worldwide (1). Accurate diagnosis and prompt treatment with appropriate antimalarial drugs are critical for reducing the malaria burden. Because of the widespread resistance of *Plasmodium falciparum* malaria parasite species to chloroquine and sulphadoxine/pyrimethamine (SP), the World Health Organization (WHO) has recommended quality-assured artemisinin-based combination therapy (ACT) for uncomplicated *falciparum* malaria since 2005 (2).

Parasite-based diagnosis is desirable before use of ACT because over-prescription of ACT, which is much more expensive than using conventional drugs, is a great threat to cost-effective intervention. Moreover, parasitological diagnosis can reduce the risk of adverse drug reactions as well as unnecessary drug pressure to malaria parasites. In most remote rural health facilities in malaria-endemic regions, however, microscopic diagnosis is limited, and malaria diagnosis has traditionally relied much on the history of fever and symptom-based diagnosis. Rapid diagnosis test (RDT) for malaria, therefore, enables accurate diagnosis in rural settings because it is easy to use, not time-consuming, and does not require electricity unlike microscopic examination (3). Consequently, in 2010, WHO changed the policy from clinical diagnosis to parasitological diagnosis, with either microscopy or RDT for all suspected malaria cases prior to treatment (4). Parasitological confirmation of malaria before treatment has been mandatory since 2015 in the latest guidelines for treatment of malaria (5).

Many studies have evaluated the impact of the introduction of RDT/ACT from health provider perspectives: reduction in antimalarial prescriptions (6–10); reduced hospital stays and prescription of antibiotics (11) and improved availability of antimalarial drugs (12). On the other hand, comparative studies of patient treatment-seeking behavior before and after the introduction of RDT/ACT have been quite few, although investigations of changes in health demand will be essential in evaluating the effectiveness of the newly introduced policy. A community-based study in Tanzania reported no significant change in health facility attendance for child fever before and after the introduction of RDT/ACT (13). In that study, however, health facility utilization for febrile children was exceptionally high (>75%) in the baseline survey partly because of a long-term social campaign at the site. Consequently, little information is available about the impact of introducing RDT/ACT on health service utilization in routine health service settings in malaria-endemic areas.

Papua New Guinea (PNG) remains a high-risk country for malaria in the Asia Pacific region. The number of malaria cases per 1,000 population was estimated to be 118 in 2015 (1). In 2011, the PNG government introduced a new protocol recommending ACT as the first-line malaria treatment together with parasite-based diagnosis with either RDT or microscopy as a result of widespread chloroquine resistance (14). By the end of the year 2012, malaria diagnosis using RDT and ACT treatment became available at the community level, including all remote/rural health facilities.

In the present study, we assessed the impact of the introduction of RDT/ACT on health demand in a rural PNG population. If patients rationally decide to maximize their utility and recognize the benefit of accurate RDT diagnosis and efficacious ACT, health demand for RDT/ACT will increase after the introduction of RDT/ACT. To prove this, we aimed to evaluate the effect of the introduction on health facility utilization to adjust for covariates using propensity score matching. Further, we investigated factors associated with health facility utilization before and after the introduction of RDT/ACT.

## Materials and Methods

### Study Area and Antimalarial Drug Supply in the Area

We conducted the study in a malaria-endemic lowland coastal area within the catchment area of a major health facility (i.e., a health center) located approximately 56 km from Wewak, the provincial capital of East Sepik Province, PNG. Malaria transmission in the study area is all-year round, and malaria is a leading cause of health facility visits. Prior to introduction of the RDT/ACT protocol, malaria was diagnosed clinically without support of microscopy at the health facility, with the antimalarial drug treatment regimen consisting of chloroquine plus SP for adults and amodiaquine plus SP for children.

Rapid diagnostic test/ACT was introduced to the formal health facilities in Wewak District in December 2011. Other than the health center, five aid posts were operated in the study site and the surrounding areas. Health center staff occasionally visited communities for a mobile clinic. There were a general hospital and two clinics in Wewak town; however, residents of the study site rarely used those facilities for malaria treatment (15). In 2007, each community assigned a village health volunteer (VHV) who clinically diagnosed malaria and provided SP plus chloroquine or amodiaquine after completion of a 1-month initial training. VHVs were allowed to use RDT/ACT after retraining in August 2012.

### Data Collection

A baseline cross-sectional survey among 20 communities was undertaken in February 2011 and February 2012. All caregivers with children aged less than 5 years were included as target interviewees. Trained field assistants interviewed caregivers to collect data on the fever episodes of their children, treatment choices, and caregiver and patient characteristics in the 2 weeks preceding the interview. If a caregiver had children aged 5–14 years, information on these children was also collected. The caregivers were primarily mothers; if not mothers, the caregivers included adult household members who mainly cared for the children, such as fathers, aunts, and grandmothers. We also obtained information on the characteristics of the health facility from direct observation or interviews with health workers. The detailed procedures of the baseline survey have been described elsewhere (15).

In February–March, 2015, a follow-up cross-sectional survey was conducted in 23 communities. The target population included all children aged less than 15 years. The same information as that in the baseline survey was collected. In addition, caregivers were asked about their knowledge of health facility locations and the experience of malaria treatment visits at health facilities in the preceding year.

### Outcome and Covariates

The outcome variable was whether caregivers of a febrile child initially chose health providers who were able to provide diagnosis and treatment in accordance with the national protocol in case of malaria (i.e., hospital, health center, aid post, clinic, mobile clinic, or VHV = 1; traditional health practitioner, pharmacy, general shop, neighbor, or self-care = 0). Covariates were selected based on our previous study (15) as follows: household’s asset index, patient’s gender, patient’s age, severity of the illness as perceived by the caregiver, the caregiver’s education, direct cost for utilization of the nearest health facility to patient’s house, distance from patient’s house to the nearest health facility, and drug availability at the nearest health facility.

In general, there was no user fee for VHV, but VHVs were allowed to charge a small amount. The observed maximum fee was PNG Kina 1 (USD 0.48 in 2011) in 2011 and 2012 and Kina 2 in 2015. In contrast, the outpatient fee for a child at formal health facilities was PNG Kina 1 (USD 0.48 in 2011) before November 2011 and Kina 2 for age <6 years and Kina 3 for age between 6 and 14 years after November 2011. This fee included the examination, a prescription, drugs, and revisit costs. The nearest health facilities for the patients were located within walking distance from their houses; thus, medical costs were equivalent to direct costs. The locations of houses and health facilities was recorded with global positioning system devices and direct distance from the house to the health-care facility was calculated using a digital map of the area (PASCO Satellite Ortho, PASCO Corporation, Tokyo, Japan) and Quantum GIS 2.14.1. To estimate asset index, seven dummy variables were selected: own mobile phone, own radio or stereo, own house with tin roof, own house with western-style wall, own generator, own rainwater tank for drinking, and own car or outboard motorboat (15). Assets were used as a proxy variable for long-term economic status by constructing a linear index of asset ownership and housing characteristics using principle component analysis (16).

### Statistical Analysis

Propensity score was estimated using a logistic regression adjusted with the covariates described above, which were possible determinants of utilization of health facilities. The vector of the covariates was defined as ***X***. Binary outcome *Y* = 1 denoted utilization of health facilities and *Y* = 0 denoted otherwise. Treatment dummy variable *Z* was assigned 1 for a treated individual, that is, a febrile child after the introduction of RDT/ACT, and 0 for a comparison individual, that is, a febrile child before the introduction of RDT/ACT. Propensity score of individual *i* was given as

$$\mathrm{Pr}(Z=1|X=X_{i})=\frac{\text{exp}(X_{i}^{\prime }\beta )}{1+\text{exp}(X_{i}^{\prime }\beta )}.$$

Each individual *i* had potential outcomes, *Y*_1_*_i_* if *Z* = 1, and *Y*_0_*_i_* if *Z* = 0; however, only one of *Y*_1_*_i_* and *Y*_0_*_i_* was observed in the study setting. Propensity score matching enabled us to estimate the missing potential outcome for each individual. We applied a full matching method: a treated individual was matched to one or more comparison individuals, with replacement, and a comparison individual was matched to one or more treated individuals with replacement. Nearest-neighbor matching was adopted within a caliper of 0.2 of the SD of the logit of the propensity score (17). We adjusted the standardized difference after matching to achieve balance of covariate (18); thereafter, average treatment effect (ATE) and average treatment effect on the treated (ATET) were estimated. Stata SE14.2 command *teffects psmatch* (StataCorp, TX, USA) was applied for the analysis. ATE and ATET were defined as:

$$\text{ATE}=E[Y_{1i}-Y_{0i}],$$

$$\text{ATET}=E[Y_{1i}-Y_{0i}|Z=1].$$

To investigate factors associated with health facility utilization, a two-level random-intercept Poisson regression model with a robust variance estimator was applied to the pooled data (19). Individual level was determined as level one and village level was applied as level two. The same variables as the covariates used for propensity score matching plus the treatment dummy variable were included in the vector of the explanatory variables ***V***. Random intercept of village *j* was defined as *u_j_*. The probability of the outcome *Y* selected by the individual *i* living in the village *j* was represented as

$$\mathrm{Pr}(Y_{ij}=y\text{|}V_{ij},u_{j})=\frac{\text{exp}(-\mu_{ij})\times \mu_{ij}^{y}}{y!}\;,\;\;y=0,1,$$

where $\mu_{ij}=\text{exp}(V_{ij}^{\prime }\beta +u_{j})$. We estimated adjusted prevalence ratio (PR) and confidence interval (CI) using Stata SE14.2 command *mepoisson*. The threshold for significance was set at *p* < 0.05 (two-tailed).

### Ethical Clearance

Ethical clearance for the study was obtained from the Medical Research Advisory Committee of the PNG National Department of Health (No. 09.26; No. 14.22) and the Tokyo Women’s Medical University Ethical Committee (No. 1744). This study was conducted in accordance with the Declaration of Helsinki and the recommendations of those committees with written informed consent from all participants.

## Results

### Descriptive Statistics

The participation proportion of the target households was 87% (736/851) in the baseline survey and 96% (1062/1103) in the survey after the introduction of RDT/ACT. A total of 4,690 children belonging to 2,143 caregivers participated in the study, and 911 (19%) fever episodes were reported in the preceding 2 weeks. Unadjusted (i.e., prematching) descriptive statistics of comparison (before the introduction of RDT/ACT) and treated (after the introduction of RDT/ACT) groups are presented in Table 1. The proportion of health service utilization increased from 52% before the introduction of RDT/ACT to 57% after the introduction, but the increase was not significant. The distributions of availability of antimalarial drugs at the nearest health facility, direct cost of the nearest facility, age of patients, and illness severity perceived by caregivers were significantly different between the comparison and treated groups.

**Table 1:** Descriptive statistics.

	Comparison group		Treated group		
	(*N* = 418)		(*N* = 493)		
Variables	*n*	%	*n*	%	*p*-Value
Health facility utilization					
Yes	216	51.7	282	57.2	0.094
No	202	48.3	209	42.4	
Missing	0	0.0	2	0.4	
Drug availability: yes	281	67.2	403	81.7	<0.001
Direct cost (PNG Kina^a^)	0^b^	0–1^c^	0^b^	0–1^c^	<0.001
Distance (km)	0.70^b^	0.26–1.18^c^	0.84^b^	0.21–1.61^c^	0.493
Age of patient (years)	4^b^	2–5^c^	5^b^	3–9^c^	<0.001
Gender of patient: male	215	51.4	257	52.1	0.834

Illness severity					
Mild	233	55.7	227	46.0	<0.001
Moderate	113	27.0	181	36.7	
Severe	40	9.6	85	17.2	
Missing	32	7.7	0	0.0	
Education of caretaker (years)	6^b^	6–8^c^	6^b^	6–8^c^	0.953
Missing	23	5.5	0	0.0	
Asset index	−0.312^b^	−0.91–0.58^c^	−0.312^b^	−0.91–0.33^c^	0.805
Missing	5	1.2	8	1.6	
Number of villages	20		22^d^		

*To compare the difference of variables between the comparison [before the introduction of rapid diagnostic test (RDT)/artemisinin-based combination therapy (ACT)] and treated (after the introduction of RDT/ACT) groups, chi square test was used for categorical variables and Wilcoxson rank-sum test was used for continuous variables*.

*^a^PNG Kina 1 = USD 0.48 in 2011 (average exchange rate calculated by the World Bank)*.

*^b^Median*.

*^c^Interquartile range*.

*^d^Of 23 villages studied, no fever episode was reported in a village*.

In 2015, about 3 years after the introduction of RDT/ACT, 99% (1165/1171) of caregivers knew the location of at least one health facility and 70% (815/1171) had visited a health facility to seek malaria diagnosis and/or treatment for their child in the preceding year.

### Estimation of ATE Using Propensity Score Matching

We excluded 7.5% (68/911) of episodes due to at least one missing value of the covariates used for matching. Consequently, 360 children from the comparison group and 483 from the treated group were included for calculating the propensity score. Although the standardized difference with respect to the gender of patients slightly exceeded 10%, the covariate balance after matching was improved (Table 2). After matching, the adjusted difference of the proportion of health service utilization after the introduction of RDT/ACT compared with the baseline proportion was positive but not statistically significant [ATE: 0.063, 95% CI −0.024 to 0.150, *p* = 0.153; ATET: 0.057, 95% CI −0.047 to 0.161, *p* = 0.283].

**Table 2:** Standardized differences of covariates.

	Standardized differences	
Covariates	Unmatched	Matched
Antimalarial drug availability: yes	0.325	0.008
Direct cost (PNG Kina^a^)	−0.235	−0.088
Distance (km)	0.092	−0.017
Age of patient (years)	0.582	−0.065
Gender of patient: male	−0.019	0.102
Illness severity: moderate	0.182	−0.005
Illness severity: severe	0.203	0.048
Education of caretaker (years)	−0.058	0.040
Asset index	−0.023	−0.043

*^a^PNG Kina 1 = USD 0.48 in 2011*.

### Factors Associated With Health-Care Utilization Using Regression Models

In line with the results of propensity score matching, the effect of the introduction of RDT/ACT on the utilization of health-care facilities was not significant using the multilevel Poisson regression model (PR, 1.07; 95% CI, 0.92–1.24) (Table 3, Model 1). Moderate- and severe-febrile patients were approximately 30 and 50% more likely, respectively, to use health facilities than mild-febrile patients (moderate: PR, 1.31; 95% CI, 1.11–1.56; severe: PR, 1.50; 95% CI, 1.22–1.87), whereas being male was inversely associated with health service utilization (PR, 0.85; 95% CI, 0.78–0.93) (Table 3, Model 1). The effect of illness severity as well as gender of patient on health service utilization was nearly consistent before and after the introduction of RDT/ACT (Table 3, Models 2 and 3). Distance to the nearest health facility was inversely associated with health service utilization in the pooled data (PR, 0.88; 95% CI, 0.84–0.93); however, the association was not significant after the introduction of RDT/ACT (Table 3, Models 1 and 3).

**Table 3:** Estimation results of multilevel Poisson model.

	Model 1: pooled data					
Fix variables	Prevalence ratio	95% Confidence interval	*p*-Value			
Treatment dummy (comparison = 0/treated = 1)	1.07	0.92–1.24	0.38			
Drug availability (no = 0/yes = 1)	1.02	0.83–1.25	0.84			
Direct cost (PNG Kina^a^)	0.99	0.83–1.18	0.90			
Distance (km)	0.88	0.84–0.93	<0.001			
Age of patient (years)	0.99	0.97–1.00	0.16			
Gender of patient (female = 0/male = 1)	0.85	0.78–0.93	<0.001			
Illness severity: moderate	1.31	1.11–1.56	0.002			
Illness severity: severe	1.51	1.22–1.87	<0.001			
Education of caretaker (years)	1.02	0.99–1.05	0.24			
Asset index	1.00	0.93–1.07	0.98			
Intercept	0.51	0.38–0.70	<0.001			
Random variable						
Village (variance of intercept)	0.01	0.00–0.10				
Number of individuals	843					
Number of villages	24					

	**Model 2: comparison group**			**Model 3: treated group**		
**Fix variables**	**Prevalence ratio (PR)**	**95% confidence interval (CI)**	***p*-Value**	**PR**	**95% CI**	***p*-Value**

Treatment dummy (comparison = 0/treated = 1)						
Drug availability (no = 0/yes = 1)	0.98	0.68–1.42	0.92	0.93	0.74–1.18	0.57
Direct cost (PNG Kina^a^)	1.24	0.90–1.71	0.19	0.91	0.78–1.06	0.21
Distance (km)	0.83	0.72–0.95	0.007	0.90	0.82–1.00	0.050
Age of patient (years)	0.98	0.95–1.01	0.11	0.99	0.98–1.01	0.42
Gender of patient (female = 0/male = 1)	0.86	0.76–0.98	0.020	0.83	0.71–0.97	0.022
Illness severity: moderate	1.32	1.06–1.64	0.013	1.32	1.07–1.62	0.009
Illness severity: severe	1.57	1.20–2.05	0.001	1.50	1.19–1.91	0.001
Education of caretaker (years)	1.03	0.97–1.09	0.30	1.02	0.99–1.05	0.25
Asset index	0.98	0.88–1.09	0.68	1.00	0.92–1.10	0.93
Intercept	0.49	0.32–0.75	0.001	0.59	0.43–0.81	0.001
Random variable						
Village (variance of intercept)	<0.001			<0.001		
Number of individuals	360			483		
Number of villages	20			22^b^		

*The data collected before and after the introduction of rapid diagnostic test/artemisinin-based combination therapy were defined as comparison and treated groups, respectively*.

*Adjusted PR and 95% CI for fix variables and variance of intercept for a random variable are shown*.

*^a^PNG Kina 1 = USD 0.48 in 2011*.

*^b^Of 23 villages studied, no fever episode was reported in a village*.

## Discussion

We have shown that (a) the introduction of RDT and ACT did not significantly affect the utilization of health facilities offering such services and (b) illness severity and gender of patient were consistent determinants of health service utilization before and after the introduction of RDT/ACT.

As a theoretical framework of access to health care, physical accessibility to health facilities and availability of good health services, financial affordability, and perceived acceptability of health services by patients are considered indispensable dimensions of health-care access (20, 21). We showed no significant effect of RDT/ACT on health service utilization with adjustments for availability, accessibility, and affordability of health-care facilities as well as patient-related individual characteristics using propensity score matching. If caregivers suspecting child malaria show a higher acceptance of RDT/ACT than of the conventional protocol, a higher utilization of health-care facilities is expected to be observed.

Incomplete information on RDT/ACT may have influenced the decision-making of caregivers. There was no active promotion of the introduction of the new protocol in PNG. Health workers informally noticed the policy change to the general public at their visits to formal health facilities. In the study area, almost all caregivers knew the location of health facilities, and a substantial proportion of them had a recent experience of malaria diagnosis and/or treatment. However, this does not mean that they had heard of the benefit and necessity of the introduction of RDT/ACT from health professionals. To increase the demand for utilization of RDT/ACT, active promotion of its importance to villagers through mass media and/or short message service may be helpful (22, 23).

Although villagers have information about the new protocol, rational decision-making is a different aspect. Several studies in African countries showed trust and positive acceptance by villagers of RDT performed by village community workers (24–26). In contrast, qualitative studies indicated that those who are familiar with conventional drugs had a negative acceptance of ACT in rural communities (27, 28). Some people in the study area may prefer chloroquine that was withdrawn from public health-care facilities. It was possible for them to get the drug at pharmacies and general shops and use it as self-medication, although it was not common to get over-the-counter drugs in the private sector (15). Investigation of villagers’ stated preferences among conventional and newly introduced protocols will be useful in formulating health promotion strategies. The management of over-the-counter drugs also needs to be considered.

The magnitude of the effect of perceived illness severity on access to health-care facilities was consistently the largest before and after the introduction of RDT/ACT, suggesting that this variable was a primary and reliable determinant of decision-making for health-care utilization. Encouragement of health facility utilization by caregivers even for their perceived mild fever in children may increase the overall use of health facilities. Systematic reviews reported that caregiver assessment of fever in children by palpation was relatively accurate in excluding fever and that its specificity was low (29, 30). Thus, a higher number of negative malaria test cases would be expected with a higher utilization proportion for perceived fever episodes. Training for VHVs on integrated fever management, including treatment for negative malaria test cases, should be required (31).

Economists have argued gender bias of parental care as unequal allocation of parental investment to maximize one’s own utility. Thomas (32) proposed the “like father, like son; like mother, like daughter” hypothesis. In the resource-limited condition, parents may make unequal allocations of resources among children. If men and women have socially different tasks, mothers may invest more in daughters and fathers in sons due to expectations of future returns to the investments in the form of help for their tasks from children of the same gender as them. If there is a conflicting interest between the father and mother about investment in a child, whether father or mother has the power to make a decision should be considered as another key determinant influencing actual behavior.

The observed gender bias of utilization of health-care facilities for febrile children may be a girl preference by female caregivers. In the study area, the division of labor based on gender and cooperation of labor between household members for food production from sago palm (*Metroxylin sagu*), the staple food of the area, were reported (33). The result was in accordance with the economic hypothesis mentioned above. However, recent analysis in 57 low- and middle-income countries reported that the proportion of utilization of health facilities for common illness of children was similar for boys and girls (34). In only two countries (Haiti and Uganda), females were more likely to be taken to health facilities, although the result from PNG was not included in the study. Because female bias in caregiver health-care-seeking behavior seemed rare (34), caution should be exercised with application of the “like father, like son; like mother, like daughter” hypothesis. Female vulnerability due to lower general health status in PNG could be a reason for the observed gender bias in health-care facility utilization (15).

Our study includes several limitations. First, propensity score matching can reduce selection bias in estimating treatment effects due to observed differences between the treatment and comparison groups, but our estimation is subject to biases from unobserved covariates. In particular, we were not able to remove the influence of an unobserved time change between 2011 and 2015 because policy change was simultaneously introduced in the study area. The global trend in treatment seeking for formal health facilities was estimated to show a 0.93 percentage point increase per year during the last 20 years (34). If this was the case in the study area, the trend increase is estimated to show a 3.7 percentage point increase before and after the introduction of RDT/ACT. Considering the magnitude of estimated ATE, a 6.3% point increase, there may be little risk of bias in the main findings because the difference of health service utilization before and after the introduction of RDT/ACT is expected to be smaller. Second, the external validity of the study was limited because our study population was limited to a catchment area of a health center in rural PNG. The proportion of health service utilization may be relatively high partly because over-the-counter use was not common in the study area. This may influence the results. Third, all information related to individual characteristics was based on caregiver reports. To minimize recall bias, we focused on fever episodes in the 2-week preceding reports.

In conclusion, we performed a propensity score matching analysis with a rural PNG population before and after the introduction of RDT/ACT to evaluate the effect of the policy change on the utilization of health facilities. The estimated ATE was not significant. The result was consistent with that of a conventional multilevel Poisson regression model. Further, we compared the factors associated with health service utilization before and after the introduction of the new malaria treatment policy. Illness severity and gender of patient were consistent determinants. Continued research in the same area will be needed to increase the internal validity of the study findings.

## Ethics Statement

Ethical clearance for the study was obtained from the Medical Research Advisory Committee of the Papua New Guinea National Department of Health (No. 09.26; No. 14.22) and the Tokyo Women’s Medical University Ethical Committee (No. 1744). This study was conducted in accordance with the Declaration of Helsinki and the recommendations of those committees with written informed consent from all participants.

## Author Contributions

TT conceived the study, designed the study, conducted the data collection, analyzed and interpreted the data, wrote the first draft of the manuscript, and completed the final version. TS designed the study and interpreted the data. TF and FH collected and interpreted the data. All authors critically revised the draft and approved the final version.

## Conflict of Interest Statement

The authors declare that the research was conducted in the absence of any commercial or financial relationships that could be construed as a potential conflict of interest. The funders of this study played no direct role in its design or execution.

## Abbreviations

ACT, artemisinin-based combination therapy; ATE, average treatment effect; ATET, average treatment effect on the treated; CI, confidence interval; GPS, global positioning system; PNG, Papua New Guinea; PR, prevalence ratio; RDT, rapid diagnostic test; SP, sulphadoxine/pyrimethamine; VHV, village health volunteer; WHO, World Health Organization.
